# Congenital pulmonary lymphangiectasia

**DOI:** 10.1186/1750-1172-1-43

**Published:** 2006-10-30

**Authors:** Carlo Bellini, Francesco Boccardo, Corradino Campisi, Eugenio Bonioli

**Affiliations:** 1 Neonatal Intensive Care Unit, Department of Pediatrics, University of Genoa, G. Gaslini Institute, Genoa, Italy; 2 Section of Lymphatic Surgery and Microsurgery, Department of Surgery, S. Martino Hospital, University of Genoa, Genoa, Italy; 3 Department of Pediatrics, University of Genoa, G. Gaslini Institute, Genoa, Italy

## Abstract

Congenital pulmonary lymphangiectasia (PL) is a rare developmental disorder involving the lung, and characterized by pulmonary subpleural, interlobar, perivascular and peribronchial lymphatic dilatation. The prevalence is unknown. PL presents at birth with severe respiratory distress, tachypnea and cyanosis, with a very high mortality rate at or within a few hours of birth. Most reported cases are sporadic and the etiology is not completely understood. It has been suggested that PL lymphatic channels of the fetal lung do not undergo the normal regression process at 20 weeks of gestation. Secondary PL may be caused by a cardiac lesion. The diagnostic approach includes complete family and obstetric history, conventional radiologic studies, ultrasound and magnetic resonance studies, lymphoscintigraphy, lung functionality tests, lung biopsy, bronchoscopy, and pleural effusion examination. During the prenatal period, all causes leading to hydrops fetalis should be considered in the diagnosis of PL. Fetal ultrasound evaluation plays a key role in the antenatal diagnosis of PL. At birth, mechanical ventilation and pleural drainage are nearly always necessary to obtain a favorable outcome of respiratory distress. Home supplemental oxygen therapy and symptomatic treatment of recurrent cough and wheeze are often necessary during childhood, sometimes associated with prolonged pleural drainage. Recent advances in intensive neonatal care have changed the previously nearly fatal outcome of PL at birth. Patients affected by PL who survive infancy, present medical problems which are characteristic of chronic lung disease.

## Disease name and synonyms

Pulmonary lymphangiectasia

Pulmonary cystic lymphangiectasis

Pulmonary lymphangiomatosis

## Definition and diagnostic criteria

Congenital pulmonary lymphangiectasia (PL) is a rare developmental disorder involving the lung and is characterized by pulmonary subpleural, interlobar, perivascular, and peribronchial lymphatic dilatation. On the basis of improved characterization of the clinical presentation and recent noteworthy advances in intensive neonatal care, the original classification [1] has been modified and PL has been sub-divided into two major categories, defined as primary and secondary PL (Tables 1, 2) [2,3].

**Table 1:** Classification of Pulmonary Lymphangiectasia

**Primary** **Pulmonary** **Lymphangiectasia**	• Primary pulmonary developmental defect • Failure of normal regression process of lymphatic channels of the fetal lung (20 weeks' gestation) • Dilated pulmonary lymphatics • Hemihypertrophy and lymphedema may be present • Syndromic (See table II)
**Secondary** **Pulmonary** **Lymphangiectasia**	• Hypoplastic left heart syndrome, pulmonary vein atresia, congenital mitral stenosis, cor triatum • Thoracic duct agenesis • Obstructive forms • Infections

**Table 2:** Autosomal dominant, autosomal recessive, and X-linked syndromes in which Pulmonary Lymphangiectasia has been described

**Syndrome**	**Inheritance**	**OMIM catalog**
Yellow nail syndrome	Dominant	#153300
Noonan	Dominant	#163950
Intestinal lymphangiectasia	Dominant	%152800
Lymphedema/cerebral arterio venous anomaly	Dominant	152900
PEHO syndrome	Recessive	%260565
German syndrome	Recessive	231080
Hennekam lymphangiectasia	Recessive	%235510
Campomelia, Cumming type	Recessive	%211890
Hypotrichosis lymphedema teleangiectasia	Recessive	#607823
Knobloch syndrome	Recessive	#267750
Urioste syndrome	Recessive	%235255
Lymphedema hypoparathyroidism	X-linked	247410
Mandibulofacial dysostosis	X-linked	--

When presenting as a primary pulmonary developmental defect, PL may be caused by a congenital defect in the primary development of the lung, or may represent the localized expression of more generalized lymphatic involvement. When it is part of generalized lymphatic dysplasia, PL presents with dilated pulmonary lymphatics as part of a generalized form of lymphangiectasia, *i.e.*, truncal lymphangiectasia, which is usually associated with lymphedema. Hemihypertrophy may also be observed, although only rarely in infants and young children.

Cardiovascular and lymphatic obstructive forms constitute the secondary PL group. Hypoplastic left heart syndrome, pulmonary vein atresia, congenital mitral stenosis, cor triatum, and thoracic duct agenesis are the most likely causes of secondary PL.

## Epidemiology

The incidence of PL is not clearly defined. Any attempt to provide precise statistics regarding the incidence of PL would be misleading considering that to date only a few isolated cases or small series have been reported. Autopsy studies suggest that approximately 0.5–1% of infants who are stillborn or die in the neonatal period have PL, and in two reported stillborn series, 5 out of 451 cases and 11 out of 2,514 cases of PL, were identified [3]. Congenital PL may be associated with non-immune hydrops fetalis and with congenital chylothorax [4]. Although the incidence of these conditions is not directly correlated to the possible incidence of PL, it may be useful to keep in mind that the incidence of hydrops fetalis in obstetric-neonatal referral centers may be as high as 1:800 [5]. Furthermore, this condition carries a poor prognosis with a mortality rate ranging from 50% to 98%, and the incidence of congenital chylothorax is about 1:10,000–15,000 pregnancies, with a male-female ratio of 2:1 [5].

## Clinical description

PL may present at birth as a stillbirth or with severe respiratory distress, tachypnea, and cyanosis, with a very high mortality rate at or within a few hours of birth [2]. Clinical diagnosis of PL can be strongly suspected in full-term neonates who present severe respiratory distress with pleural effusion (especially if chylous) at birth, with or without generalized or localized lymphedema.

As reported in the early studies on this topic, before effective mechanical ventilation became available most children did not survive. Mechanical ventilation has almost always been required in the most recently reported cases [2,3].

In the post-neonatal period, children with PL present with respiratory difficulties of varying degree, associated with a relapsing course. During both the neonatal and post-neonatal period, PL may be associated with chylothorax, chylopericardium, and chylous ascites. In older children it is frequently associated with recurrent cough, wheeze, increased respiratory effort with inspiratory crackle, and even congestive heart failure. The disease is characterized by frequent respiratory exacerbations [3,6].

## Etiology

The etiology of PL is not known. It has been suggested that PL lymphatic channels of the fetal lung do not undergo the normal regression process at 20 weeks of gestation, and thus there is a persistence of the large lymphatic vessels that are normal form of the maturation developmental process at 9–16 weeks of gestation [7]. Obstruction of pulmonary lymphatics or veins, or the action of infectious agents have also been taken into consideration [2].

Secondary PL may be caused by a cardiac lesion. Pulmonary lymphatics dilatation develops *in utero *as a result of obstructed pulmonary venous flow, or cardiac lesions which have been hypothesized to interfere with the normal regression of the lymphatic tissue elements after the 16th week of fetal life [3].

Lymphangiogenesis has been reviewed in several recent papers [8-11]. Lymphatic vessels were first described in the seventeenth century by the Italian scientist G. Aselli. However, until a few years ago, the lymphatic system was not considered an interesting topic. In 1902, Florence Sabin proposed the most widely accepted model of lymphatic vasculature development. According to Sabin's proposal, the peripheral lymphatic system originates from the primary lymph sacs (jugular lymph sac, subclavian lymph sac, retroperitoneal lymph sac, cysterne chyli, posterior lymph sac), then spreads by endothelial sprunting into the surrounding tissue and organs, where local capillaries are formed. A less popular model was proposed in 1910 by Huntington and McClure, who claimed that primary lymph sacs arise in the mesenchyme independently of the veins and then establish venous connections. This model has recently been supported by a study on chickens. On the basis of recent studies, it has been proposed that mammalian lymphatic vasculature develops in four stages: lymphatic endothelial cell (LEC) competence, LEC bias, LEC specification, and LEC differentiation. Knowledge about the genes that are expressed and/or required during lymphatic vasculature formation and about the signal-transducing system for lymphatic endothelial cell growth, migration, and maturation has improved significantly.

**Figure 1:**
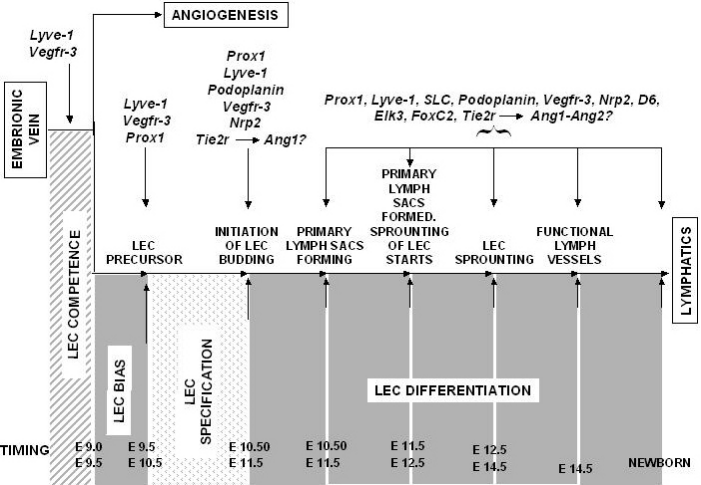
**Molecular pathways in Lymphangiogenesis**. The four-step model for lymphatic vasculature formation is summarized in this scheme. Time line of endothelial cell gene expression in lymphangiogenesis refers to the murine lymphatic system. **LEC competence **is the autonomous ability of venous endothelial cells to respond to an inductive signal. **LEC bias **is the bias toward LEC determination and is characteristic of LEC precursors; LEC bias is eventually lost in other venous endothelial cells. **LEC specification **is characterized by initiation of LEC budding; LEC specification occurs when biased endothelial cells differentiate. **LEC differentiation **is characterized by four different events, as shown in the figure. During this period lymphatic vessel differentiate and maturation occurs in a stepwise manner leading to the syntesis of all of the main lymphatic vessel components to mature lymphatics. LEC: lymphatic endothelial cell. Timing: E means embryonic days. *Lyve1*: lymphatic vessel endothelial hyaluronan receptor 1. *Vegfr3*: vascular endothelial growth-factor receptor 3. *Prox1*: prospero related homeobox 1. *Nrp2*: neuropilin 2. *Tie2*: endothelial cell-specific receptor. *Ang1 *and *2*: angiopoietin 1 and 2. *SLC*: secondary lymphoid chemokine. *D6*: **β**-chemokine receptor. *ELK3*: ETS-domain protein (SRF accessory protein 2). *FOXC2*: forkhead box C2.

Table 3 and Figure 1 summarize the most up-to-date information regarding molecular pathways in lymphoangiogenesis and on lymphangiogenetic markers. Table 3 and Figure 1 have been obtained, with modifications, from data published in previously cited papers [8-11].

**Table 3:** Lymphangiogenesis: genes involved in lymphatic vasculature formation

**Gene**	**Function**	**Expression and function**	**Lymphatic phenotype**
*Ang2*	Growth factor, ligand of Tie-2	Lymphatics express Tie receptor family members. Smooth muscle cells of large vessels. The endothelium of smaller vessels at sites of vacular remodelling induces its expression.	Hypoplasia, chylous ascites
D6	**β**-chemokine receptor D6	It may play a role in chemokine-driven leukocyte recycling through the lymphatics.	No available animal model
*FoxC2*	Forkhead/winged-helix transcription factor	Paraxial, presomitic mesodermic and developing somites. Later restricted to condensing mesenchyme of the vertebrae, head, limbs, and kidney.	Abnormal lymphatic pattern, absent valves, lymphatic vessel and lymph node hyperplasia. It is related to lymphedema-distichiasis
*Elk3 *(Net)	Transcription factor	Expressed by the embryonic and adult vasculature: LECs and muscle layer in the thoracic duct, intestine and skin lymphatic vessels in mid-gestation.	Lymphangiectasis, chylothorax
*Lyve1*	Lymphatic vessel endothelial hyaluronan receptor-1 (CD44 homolog)	Embryonic and adult LECs. Hypothesized to function in the transport of hyaluronan.	Not available
*Nrp2*	Non-tyrosine kinase receptor for VEGF165, VEGF145, PIGF, VEGF-C and class 3 semaphorins	Embryonic and adult LECs.	Transient hypoplasia of lymphatic capillaries.
Podoplanin	Membrane glycoprotein	Embryonic and adult LECs.	Lymphangiectasis, abnormal lymph transport, lymphedema. Respiratory failure due to abnormal lung development.
*Prox1*	Homeobox transcription factor	Embryonic and adult LECs. Required for further differentiation of lymphatic "biased" cells to the fully differentiated form	No lymphatic vessels, chylous ascites, adult onset obesity.
*SLC*	?	Embryonic and adult LECs	Not available
*Vegfr3*	Receptor tyrosine kinase that mediates VEGF-C/D	BECs and LECs early development, down-regulated by BECs, but remains high in LECs later during embryogenesis.	Hypoplasia, chylous ascites. It is related to Milroy disease
VEGFC	Growth factor, ligand of VEGFR3	Mesenchymal cells around embryonic veins, activated macrophages, skeletal muscle cells, and smooth muscle cells surrounding large arteries.	No lymphatic vessels, hypoplasia, chylous ascites
*Tie2*	Receptor for *Ang1 *and *Ang2*?	Embryonic and adult LECs?	Lymphatic defect

*Ang1*: angiopoietin. *D6*: **β**-chemokine receptor. *ELK3*: ETS-domain protein (SRF accessory protein 2). *FOXC2*: forkhead box C2. *Lyve1*: lymphatic vessel endothelial hyaluronan receptor 1. *Nrp2*: neuropilin 2. *Prox1*: prospero related homeobox 1. *SLC*: secondary lymphoid chemokine. *Tie2*: endothelial cell-specific receptor. *Vegfr3*: vascular endothelial growth-factor receptor 3. *VegfrC*: vascular endothelial growth-factor receptor C. LEC: lymphatic endothelial cell. BEC: blood endothelial cell.

## Diagnostic methods

As previously reported, during the prenatal period all the causes leading to hydrops fetalis have to be taken into consideration in the diagnosis of PL [12-14]. The diagnostic approach includes the following: complete family and obstetric history; ultrasound examination and magnetic resonance (MR) studies searching for twin gestation, anatomic abnormalities, heart fetal echo and doppler blood flow assessment; maternal evaluation including blood type, Rhesus factor (Rh), antibody screening, Kleihauer-Betke stain, TORCHES-CLAP titer (Toxoplasma gondii; Rubella virus; Cytomegalovirus; Herpes simplex virus; Enterovirus; Syphilis; Chickenpox [varicella-zoster] virus; Lyme disease [borrelia burgdoferi]; Aids; Parvovirus B19), metabolic studies, and hemoglobin (Hb) electrophoresis; invasive fetal assessment involving amniocentesis (karyotype, cultures, TORCHES-CLAP, and restriction endonuclease and fetal blood sampling: blood type, Hb) electrophoresis, blood gas, cultures, TORCHES-CLAP, and DNA analysis, and fetal effusion sampling (TORCHES-CLAP, protein content, and cell count) [14].

The postnatal diagnostic approach includes a laboratory and instrumental evaluation that is needed to rule out various conditions possibly related to PL, and to establish whether PL is primary or secondary.

Hematologic causes can be ruled out by blood cell count, Kleihauer-Betke stain, Hb electrophoresis, cell blood count (CBC) and smear; cardiovascular causes can be excluded by echocardiogram and electrocardiogram (ECG); congenital infections by TORCHES-CLAP titer evaluation. Genitourinary causes can be excluded by kidney sonography, blood urea nitrogen (BUN), and plasma-urine creatine analysis. Lastly, chromosomal, syndromic, and metabolic diseases need to be ruled out by the usual diagnostic protocols.

**Figure 2:**
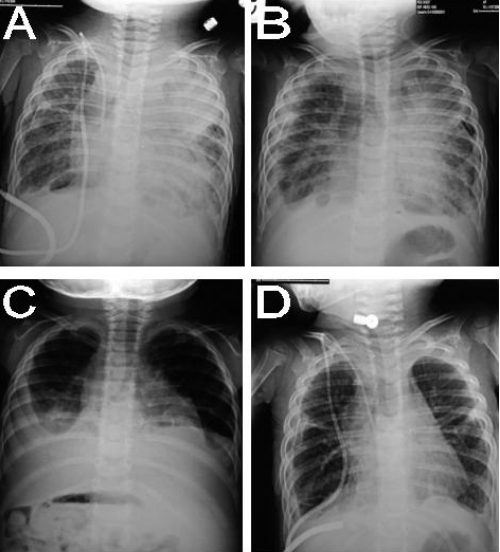
**Pulmonary Lymphangiectasia. Chest radiographs, AP views**. Radiological findings occurring during the clinical course of PL. A and B: over time progression of hazy perihilar infiltrates on the left lung. C: important bilateral pleural effusion. D: after pleurodesis, bilateral lung hyperinflation with interstitial and septa thickening are evident, and a mild degree of pleural effusion remains.

**Figure 3:**
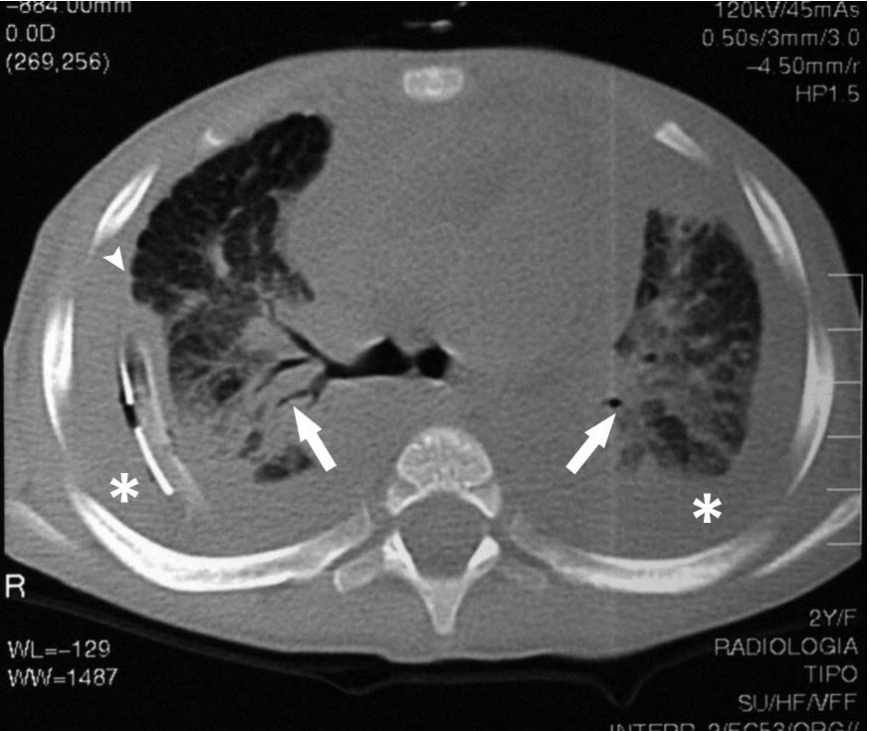
**Pulmonary Lymphangiectasia. High-resolution computed tomography (HRCT)**. Diffuse thickening of the peribronchovascular interstitium and the interlobular septa (arrowheads), associated with bilateral pleural effusion (*), and peribronchovascular infiltrates (arrows) with bronchogram.

**Figure 4:**
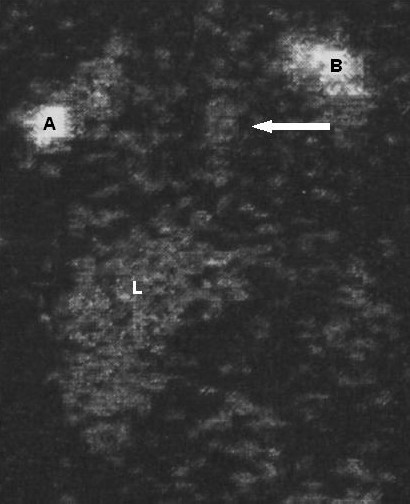
**Lymphoscintigraphy**. Lymphoscintigraphic study of a patient affected by pulmonary lymphangiectasia and generalized lymphedema showed signs of dermal back-flow in the right lower limb. A and B = patient's hands. L = liver. Arrows point to the thoracic duct.

Diagnostic methods (Table 4) that may be useful in evaluating PL include conventional radiologic studies (Figure 2), high-resolution computed tomography (CT) (Figure 3) and MR imaging [2,4,6,15-26], lymphoscintigraphy (figure 4) [2,4,26,27], lung functionality tests [6,12], lung biopsy [6,19], bronchoscopy [6,19], and pleural effusion examination [28-30]. Chest x-rays usually show hyperinflation with interstitial markings.

**Table 4:** Pulmonary Lymphangiectasia: diagnostic work-up

**Diagnostic test**	**Main features**	**Comment**
Chest x-ray	Hyperinflation with interstitial markings	Radiological findings in PL may improve over time. Longitudinal follow-up pointed to the possible progression of hazy infiltrates, that are usually seen during the neonatal period, to a more perihilar interstitial pattern with varying degrees of lung inflation
High Resolution Computed Tomography (HRCT)	Diffuse thickening of the interstitium, both of the peribronchovascular interstitium and the septa surrounding the lobules	HRCT is the technique of choice for diagnosing PL
Magnetic Resonance Imaging (MRI)	Coronal MRI T1 may permit to show thickening of the interstitium, pleural fluid effusion, and atelectasia. Axial MRI T2 usually shows high-signal material within the pulmonary interstitium, which is very often associated with pleural effusion.	HRCT is better than MRI not only in diagnosing PL, but, more in geeral, for the diagnosis of pediatric interstitial lung disease.
Lung biopsy	Useful for demonstrating the presence of dilated lymphatic spaces in the sub-pleural connective tissue, along thickened interlobar septa, and around bronchovascular axes	Great caution must be taken when preparing histological specimens and when interpreting lung biopsies or autopy samples
Lymphoscintigraphy	Useful for evaluating lung lymph vessel involvement by showing radiotracer accumulation in the lung and by providing evidence of back-flow within the thoracic duct	It provides valuable morpho-functional information regarding the lymphatic system
Bronchoscopic evaluation and lung function tests	Not specific	They may be useful for ruling out other pulmonary pathologies and for carrying out bronchial lavage in order to identify and isolate respiratory pathogens

Longitudinal follow-up indicates the possible progression of hazy infiltrates, which are usually seen during the neonatal period, to a more perihilar interstitial pattern with varying degrees of lung inflation. Generally speaking, it may be affirmed that, like the clinical features, the radiological findings in PL improve over time.

CT demonstrates diffuse thickening of the interstitium, both of the peribronchovascular interstitium and the septa surrounding the lobules. Helical chest CT usually highlights eventual diffuse interstitial change involving thickening of the interlobular septa, which is very often associated with the presence of pleural fluid effusion and atelectasia.

Findings from CT scan studies have also shown improvement over time, although the regional pattern of parenchymal inhomogeneity persisted in several studies.

Coronal MRI T1 may show thickening of the interstitium, pleural fluid effusion, and atelectasia, if present. Axial MRI T2 usually shows high-signal material within the pulmonary interstitium, which is very often associated with pleural effusion.

It has been demonstrated that despite the greater dose of radiation that is given during CT as compared to chest radiography, CT is preferable for the diagnosis of PL and, more generally, for the diagnosis of pediatric interstitial lung disease.

Lymphoscintigraphy is a mildly invasive technique that provides valuable morpho-functional information regarding the lymphatic system. It highlights the accumulation of lymphatic fluid in the interstitial tissue that causes swelling, which is most evident in the limbs. Lymphoscintigraphy is useful for evaluating lung lymph vessel involvement by proving there is an accumulation of radiotracer in the lung and by providing evidence of back-flow within the thoracic duct.

It is also useful for evaluating possible generalized, associated lymph vessel dysfunction by revealing delay, asymmetric or absent visualization of regional lymph nodes, "dermal back-flow", asymmetric visualization of lymphatic channels, collateral lymphatic channels, interrupted lymphatic structures, and lymph nodes of the deep lymphatic system.

Borderline disease may occur in the newborn. In these cases, quantitative analysis, obtained by determining the transport index, may increase the sensitivity and specificity of lymphoscintigraphy in the very early diagnosis of lymphatic disorders of the newborn.

Evaluation of pleural effusion: chylothorax is usually diagnosed in the presence of pleural effusion with a triglyceride level >1.1 mmol/L and a cell count >1,000 cells/**μ**L, with a predominance of lymphocytes (approximately 80%), according to the criteria drawn up in previous reports. However, this is an unreliable diagnostic test in malnourished patients and in patients not receiving enteral nutrition, including the fetus and occasionally the neonate. Without enteral feeding, not enough chylomicron (the main triglyceride carrier) is produced to raise chyle triglyceride levels. In these patients, a diagnosis of chylothorax may easily be made by detecting lymphocytes in the pleural fluid.

In the few cases in which lung function tests were performed, they showed various patterns including restrictive, obstructive, and normal values. It is noteworthy that pulmonary function tests were stable over time in the patients who obtained multiple values.

Bronchoscopic evaluation, while not specifically indicated in PL, may be useful for ruling out other pulmonary pathologies, and for performing bronchial lavage to identify and isolate respiratory pathogenic organisms. No tracheo-bronchial anatomical abnormalities were reported in PL patients who were evaluated by bronchoscopy. Signs of bronchitis are often reported.

Lung biopsy may be useful to demonstrate the presence of dilated lymphatic spaces in the sub-pleural connective tissue, along the thickened interlobar septa, and around the bronchovascular axes.

Great care must be taken when interpreting lung biopsies. In fact, the pathological findings in PL patients may change a great deal over time, especially in case of viral infection, and, more generally, may span from initial recognition of minimal evidence of lymphatic dilatation, possibly related to a technical artifact (cross-clamping of the lung), to proof of severe lymphangiectasia. In this case, the lymphatic vessels are characterized by a thin wall, devoid of smooth muscle, and with slightly dilated lumen, lined by flattened endothelial cells. However, severe clinical conditions frequently do not allow lung biopsies to be performed, especially in newborns.

Post-mortem examinations of the lung may be difficult and sometimes not very informative. Lung removal during autopsies causes the lymphatics to collapse, thus preventing the network of intercommunicating channels from being evaluated. It is occasionally difficult to differentiate PL from lymphangiomatosis by histological examination because both conditions have similar clinical manifestations and the histology is occasionally similar [31]. Pathological features of lymphangiomatosis include a proliferation of complex anastomosing lymphatic channels that markedly expand the typical lymphatic routes within the lungs and mediastinum. Compared to PL, there is a prominence of collagen and spindle-shaped cells surrounding the endothelia cell lined channels in lymphangiomatosis. In addition, in lymphangiomatosis there is an increased number of dilated lymphatic channels, whereas in PL the lymphatics are not increased in number. Pleural effusions are common in lymphangiomatosis. In addition, the evaluation of the affected tissues under the microscope with the standard staining techniques (hematoxylin and eosin), it has been reported that immunohistochemical staining is useful; antibodies D2-40 and CD 31 are excellent lymphatic endothelium markers [32]. PL has also been associated with multiple congenital anomaly syndromes, including Noonan syndrome, Turner syndrome, Down syndrome [33-35], Frijns syndrome [16], Hennekam syndrome [4,36], Milroy syndrome [16], and Urioste syndrome [33,34,37]. A syndromic classification of hereditary lymphedema has been recently proposed [38].

## Differential diagnosis

PL may be diagnosed during the prenatal and/or neonatal period, or in older children or adults when it presents with a milder course [2,3,6,39,40].

During the prenatal period, all causes leading to hydrops fetalis have to be taken into consideration. Hydrops is the end stage of a variety of diseases, and is reached through three primary mechanisms *i.e.*, congestive heart failure, decreased plasma osmotic pressure, and obstructed lymphatic flow [14].

During the neonatal period, transient tachypnea of the newborn, pulmonary aspiration syndrome and interstitial pulmonary infection are well known and are usually taken into consideration in the differential diagnosis of respiratory distress syndrome in the neonate. Rarer conditions must also be considered in the differential diagnosis of chronic interstitial lung disease in infants, and include surfactant protein B deficiency, desquamative interstitial pneumonitis (familial and non-familial forms), pulmonary alveolar proteinosis, idiopathic pulmonary fibrosis, lymphoid interstitial pneumonitis, cellular interstitial pneumonitis, and chronic pneumonitis of infancy. Moreover, other conditions that can mimic interstitial lung disease in infants and children should be considered. These include persistent tachypnea of infancy, neuroendocrine cell hyperplasia of infancy, acute pulmonary hemorrhage of infancy, follicular bronchiolitis, pulmonary vascular disorders (obstructive pulmonary venous disease, *i.e.*, total and partial anomalous pulmonary venous return, pulmonary vein atresia or stenosis), hereditary hemorrhagic teleangiectasia, pulmonary hemangiomatosis, various systemic diseases, and metabolic lipid storage disorders.

Finally, PL should be taken into consideration in the differential diagnosis of children and adults with chronic respiratory symptoms and rare interstitial lung diseases, including cystic fibrosis, gastroesophageal reflux, ciliary disorders, collagen vascular disorders, pulmonary hemosiderosis, immunodeficiency syndromes, or hypersensitivity peumonitis. It must be kept in mind that many of the disorders listed above as pertaining to the neonatal period may also have a later onset in childhood or even in the young adult.

It must be said that chronic interstitial lung disease is a challenging diagnostic clinical problem, which requires a systematic and multidisciplinary approach.

## Genetic counseling

The low number of reported cases does not permit consistent genetic counseling to be performed. Most cases are sporadic. Affected siblings have been described both in cases of the isolated primary form, and occasionally in various genetic multiple congenital anomalies. Male predominance in the primary form is reported, but data are not entirely convincing.

In a recent review [38], pulmonary lymphatic dysplasia was described in

• autosomal dominant syndromes: Yellow nail syndrome – OMIM #153300, Noonan – OMIM #163950, Intestinal lymphangectasia – OMIM %152800, Lymphedema/cerebral arterio-venous anomaly – OMIM 152900;

• autosomal recessive syndromes: PEHO syndrome – OMIM %260565, German syndrome – OMIM 231080, Hennekam lymphangectasia – OMIM %235510, Campomelia, Cumming type – OMIM %211890, Hypotrichosis lymphedema teleangectasia – OMIM #607823, Knobloch syndrome – OMIM #267750, Urioste syndrome – OMIM %235255;

• X-linked syndromes: Lymphedema hypoparathyroidism – OMIM 247410, Mandibulofacial dysostosis, lymphedema syndrome [41].

The occurrence of PL in siblings, and the association with a wide number of autosomic recessive syndromes would make a recessive mode of inheritance a reasonable hypothesis, although to date this has not been proved. When PL occurs as part of other known syndromes, such as Down syndrome, Noonan syndrome, or other previously mentioned conditions, genetic counseling should refer to the common recommendations that are usually made for each known syndrome [33-37,42,43].

## Antenatal diagnosis

Obstetric fetal ultrasound evaluation plays a key role in the antenatal diagnosis of PL.

The conditions leading to a pathologic increase in interstitial and total fetal body water may be correlated to congenital PL, and, more generally, should be correlated to conditions that cause hydrops fetalis. Hydrops fetalis must be taken into consideration in the presence of generalized skin thickening of >5 mm, and two or more of the following signs: placental enlargement, pericardial effusion, pleural effusion, or ascites [5].

Any of these conditions may occur in cases of congenital PL. Most studies on fetal hydrops, however, include cases in which fluid accumulation is not present in all compartments. It is generally assumed, in fact, that the etiology is the same and that these cases represent an earlier stage of the same pathological condition.

Although this is true for some pathologies, others lead to an accumulation of fluid only in some compartments (*i.e.*, isolated ascites, isolated hydrothorax, or other isolated conditions), without clear progression to generalized hydrops. Very early ultrasound recognition of abnormal fluid accumulation often ends in premature birth, thus generalized hydrops cannot fully develop. This may generate considerable overlap in the literature among hydrops, nuchal cystic hygroma, and accumulations of lymph fluid in body cavities caused by dysplasia and/or obstruction of lymphatic vessels [5].

## Management including treatment

Treatment is generally supportive. At birth, in the presence of severe respiratory distress associated with pleural effusion, delivery room management could be a challenge and multiple procedures might be required. Tracheal intubation and assisted ventilation are usually necessary. When effective gas exchange is not achieved, sterile thoracentesis and/or paracentesis should be considered. Fluid replacement, inotropic support and, in case of persistent pulmonary hypertension, ventilatory management with high frequency oscillatory ventilation and/or nitric oxide may be necessary. Airway, chest wall, and pulmonary edema, pleural effusion, pulmonary hypoplasia with associated respiratory distress syndrome, perinatal depression, hypoxia, and acidosis are the main problems that occur during delivery room resuscitation, and then during birth stabilization.

The immediate, at birth evacuation of the pleural effusion with assisted ventilation may lead to a favorable outcome of respiratory distress.

Respiratory problems that occur in the post-neonatal age, and that can continue over the next years of life, often require home supplemental oxygen and symptomatic treatment for recurrent cough and wheeze. A great deal of attention must be paid to avoid bronchitis, since common respiratory pathogens are usually involved. Cultures from bronchoalveolar lavage should be done in order to start selective antibiotic treatment.

In patients with rapidly expanding pleural effusion that requires placing unilateral or bilateral chest tube(s), the large amount of fluid (that is drained over days and weeks) leads to the loss of great quantities of albumin, immunoglobulin, and many other plasma factors that must be replaced, in some cases even on a daily basis. Gastroesophageal reflux requires standard treatment.

Nutrition plays an important role in reducing lymphatic production. Enteral nutrition with medium-chain triglycerides and total parenteral nutrition have been successfully employed [2,3].

Octreotide and antiplasmin have been used in PL and in intestinal lymphangiectasia. Non-univocal data are available regarding the effectiveness of these drugs [44-48].

When the chyle leakage persists (intractable chylothorax), pleurodesis by instillation of sclerosing agents (talc, fibrin glue, povidone-iodine) or parietal pleurectomy appear to be effective. Pleurodesis may be associated with thoracic duct ligation or suture of leaking collaterals [49-51].

## Prognosis

Contradictory data have been reported regarding the outcome. A recently reported series [6,17] stated that respiratory symptoms improved over time in the majority of patients (8/9), including those who presented in the neonatal period (3/9). These data are in contrast with those from a previously reported 11 patient series [17], in which all patients who had been diagnosed during the neonatal period died (6/11). It must be pointed out that in this latter study, 2/11 patients were born at <30 weeks' gestation, and that another 4/11 subjects had complex cardiac abnormalities. In the former study, the occurrence of cardiac involvement was less severe, and included pulmonary stenosis in 2/9 patients, and mild tricuspid regurgitation in 3/9, including one patient who also presented pulmonary hypertension. When diagnosis is made in childhood or adult age, the outcome is more likely to be favorable *quoad vitam *[6].

Other small series and single case reports do not allow a consistent prognosis to be established [2-4,15-18,35,43,52-56].

Recent advances in intensive neonatal care have changed the previously nearly fatal outcome of PL at birth. Patients affected by PL who survive infancy often present medical problems that are characteristic of chronic lung disease. Gastroesophageal reflux and poor growth are also not uncommon during the first year of life, especially between six and twelve months of age, and are closely related to chronic lung disease. If chylothorax occurs, a number of components are lost, including fats (mainly phospholipids, cholesterol, and triglycerides), proteins (mainly albumin, immunoglobulins, and fibrinogen), electrolytes, and fat-soluble vitamins in concentrations similar to those found in plasma, thus causing severe manifestations of severe deficiency.

## Unresolved questions

A better understanding of the molecular etiology of PL may be reached through improved knowledge of the early steps of lymphatic endothelial cell differentiation, novel mechanisms of lymphatic vascular growth, maturation of lymphatic vessels, and functional genomics of the lymphatic versus blood vasculature.
